# A Wireless Sensor Network with Soft Computing Localization Techniques for Track Cycling Applications

**DOI:** 10.3390/s16081043

**Published:** 2016-08-06

**Authors:** Sadik Kamel Gharghan, Rosdiadee Nordin, Mahamod Ismail

**Affiliations:** 1 Department of Electrical, Electronic and Systems Engineering, Faculty of Engineering and Built Environment, Universiti Kebangsaan Malaysia, UKM Bangi, Selangor 43600, Malaysia; adee@ukm.edu.my (R.N.); mahamod@ukm.edu.my (M.I.); 2 College of Electrical and Electronic Engineering Techniques, Middle Technical University, Al Doura 10022, Baghdad, Iraq

**Keywords:** cycling, distance estimation, optimization technique, soft computing, WSN

## Abstract

In this paper, we propose two soft computing localization techniques for wireless sensor networks (WSNs). The two techniques, Neural Fuzzy Inference System (ANFIS) and Artificial Neural Network (ANN), focus on a range-based localization method which relies on the measurement of the received signal strength indicator (RSSI) from the three ZigBee anchor nodes distributed throughout the track cycling field. The soft computing techniques aim to estimate the distance between bicycles moving on the cycle track for outdoor and indoor velodromes. In the first approach the ANFIS was considered, whereas in the second approach the ANN was hybridized individually with three optimization algorithms, namely Particle Swarm Optimization (PSO), Gravitational Search Algorithm (GSA), and Backtracking Search Algorithm (BSA). The results revealed that the hybrid GSA-ANN outperforms the other methods adopted in this paper in terms of accuracy localization and distance estimation accuracy. The hybrid GSA-ANN achieves a mean absolute distance estimation error of 0.02 m and 0.2 m for outdoor and indoor velodromes, respectively.

## 1. Introduction

One of the key challenges in wireless sensor networks (WSNs) is localization [1]. Localization of sensor nodes in WSNs is essential since it reflects spatial context with the data gathered by sensor nodes and used in applications [2]. There are several applications related to location knowledge in WSNs, such as target tracking [3], person tracking, monitoring [4], unmanned aerial vehicles [5], patient fall detection [6], wild forest areas [7], agriculture [8], disasters [9], and environment management [10]. Given the various constraints in WSN networks, accuracy is the main challenge in WSN localization techniques [11]. These techniques differ based on physical location (e.g., *x* and *y* coordinates in a two-dimensional Cartesian map or distance between nodes); types of environment (e.g., outdoor or indoor); network topology (e.g., centralized, decentralized, or remote sensing); and wireless protocol and cost (e.g., Bluetooth, Wi-Fi, ZigBee, or RF). Sensor nodes (SNs) in most WSN applications are powered by a battery and the amount of energy consumed by the nodes determines the network’s lifespan. For future Internet of Things (IoT) applications, reducing energy consumption and prolonging the battery life of SNs have become compulsory. The power consumption of sensor nodes can be reduced by controlling the transmitted power of the wireless protocols. This control can be achieved through an accurate distance estimation between the nodes in the WSN. One method of reducing sensor node power consumption is by exploiting the location or distance between the nodes.

**Figure 1:**
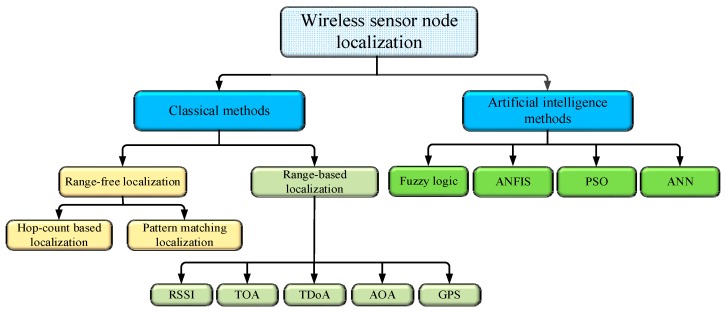
Classification of localization techniques.

There are several techniques used in WSN localization. These techniques can be classified into two categories: classical localization techniques and artificial intelligence techniques that use soft computing optimization algorithms, as shown in Figure 1. Classical localization techniques have been introduced in previous research works and include range-based and range-free. The range-based method can be used to estimate the distance or angle between sensor nodes. This method has a high level of accuracy but requires extra hardware. Examples of this approach is time of arrival (ToA) [12], phase of arrival (POA) [13], angle of arrival (AoA) [14], time difference of arrival (TDoA) [15], received signal strength (RSS) [16], acoustic energy [17], and global positing system (GPS) [18]. In contrast, the range-free method has a low level of accuracy for the estimation of the sensor node location while being cost-effective [19]. It depends on the connectivity between fixed nodes, known as anchor nodes, and a further mobile or stationary sensor node. The range-free method determines the position of the sensor node without achieving distance estimation [20]. Examples of this approach are the centroid localization technique [21], weighted centroid localization technique [22],hop-count-based localization [23], and the pattern matching method [24]. Artificial intelligence localization techniques have been used in previous research, including Artificial Neural Network (ANN) [25], Neural Fuzzy Inference System (ANFIS) [26], Fuzzy logic [27], and optimization algorithms, such as Genetic Algorithms [28], Particle Swarm Optimization (PSO) [29], Bacterial Foraging Algorithm (BFA) [30], and Gravitational Search Algorithm (GSA) [31].

However, in most previous localization techniques, the localization or distance accuracy is still not satisfactory. The distance estimation accuracy of track cycling is essential for paving the way for more studies related to energy saving in WSNs, where the transmitted power of the sensor nodes can be controlled according to the accurate distance measurement between the coach and the bicycle on the track. With accurate localization, the power consumption of the WSN can be improved and the battery life of the sensor nodes can be extended. This paper presents range-based localization using RSSI with two soft computing techniques (i.e., ANFIS and ANN) to improve the distance estimation accuracy between the mobile node (i.e., bicycle) while moving on the cycle track and the coach (located at the centre of the track cycling field) in both outdoor and indoor velodromes. Three optimization algorithms PSO, GSA, and Backtracking Search Algorithm (BSA), were combined individually with ANN to improve the distance estimation accuracy. The PSO, GSA, and BSA were designed to determine the optimum number of neurons in each hidden layer and the learning rate of ANN. A high level of distance estimation accuracy was achieved that was better than in the previous works.

## 2. Related Cycling Localization Techniques

Several studies adopting different approaches for wireless positioning on a bicycle track have been conducted. The location and speed of an athlete were monitored in [32] using GPS as part of a server-based mobile coaching system (MCS). An MCS was used in the Advanced and Adaptive Network Technology (ANT) wireless protocol to monitor the biomechanical and physiological parameters of the bicycle and the cyclist, respectively. In research work [33], a remote mobile monitoring system (RMMS) was implemented using a ZigBee WSN. This RMMS was able to monitor physiological and biomechanical parameters using several sensors that transmitted their data to the coordinator node, which communicated with a small laptop that was fixed to the bicycle handle bar. Bicycle location was estimated using GPS, which was installed on the bicycle laptop. Location information was sent to a remote server to be monitored by a remote user.

In [34], the authors combined a wheel sensor and a compass sensor to determine bicycle location instead of using GPS in order to reduce the cost and the power consumption of the bicycle area network (BAN). In particular, a bicycle may be used in places other than common cycling environments (for example in dense forests and between tall buildings in a city), where GPS is not useful [34]. However, this method is not sufficiently accurate to use in place of GPS. Zhan et al. [35] used GPS and a cellphone to track bicycle trips in outdoor environments. The proposed system saved a significant amount of energy (57%) by adjusting the duty cycling of the GPS. A similar approach (i.e., GPS) was also used in several studies on cycling positioning [36,37,38]. However, the GPS method is inefficient for indoor positioning, although it is useful in outdoor environments when LOS between a satellite and a receiver is available without any barriers [39]. In addition, GPS methods are affected by several factors that result in high localization errors of 1–30 m [40]. Moreover, GPS has other limitations, such as a poor battery life, and it is very expensive [41] when deployed in large quantities.

Wireless bicycle sensor nodes with a Wi-Fi infrastructure have been proposed by Pias et al. [42] to estimate bicycle location in Cambridge city centre (2.5 km × 2.5 km). The proposed system consisted of three subsystems: mobile nodes, the central server, and the communication infrastructure. The mobile nodes communicated with the trackside servers, which in turn transferred the data to the central server to determine bicycle location on the track. However, localization with Wi-Fi is an expensive and impractical method that requires a huge infrastructure, such as a large number of Wi-Fi clients, routers and gateways in a city.

Shin et al. [43] used a ZigBee WSN to monitor the position of a cyclist on a cycle path. A path length of approximately 13 km was considered in their study. A total of 30 wireless router nodes were distributed along the cycle path. The router nodes along the path constantly recorded the bicycle position and the biomedical parameters of the cyclist. Bicycle location was determined based on the communication between the bicycle node and the router nodes. The router nodes transmitted the unique Medium Access Control (MAC) address of the bicycle and the router ID to the server through a gateway, whereby the latitude and longitude of the routers and the gateways were known in advance and recorded in the database of the server. Thus, the router node should be aware of the position of the bicycle, which would be recorded to determine bicycle location on the path. The authors stipulated that approximately 18 s would be required to transfer the data from the bicycle to the server because of the considerable number of routers along the path. During this time delay, the bicycle could communicate with the next router, and the location of the bicycle would be recorded in the database along with that of the previous router. Consequently, a positioning error would occur. Because of the aforementioned challenges and limitations for localization accuracy, the proposed soft computing techniques based on optimization algorithms (i.e., PSO, GSA, and BSA) were introduced in this research to improve the distance estimation accuracy of the bicycle moving along the cycle track.

## 3. Mobile Node System Model

**Figure 2:**
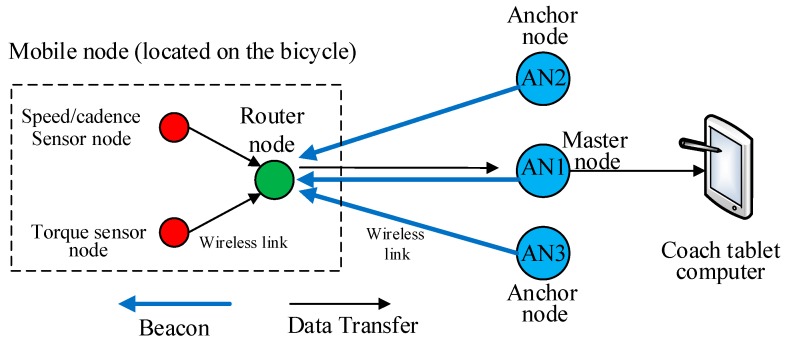
BWSN topology.

**Figure 3:**
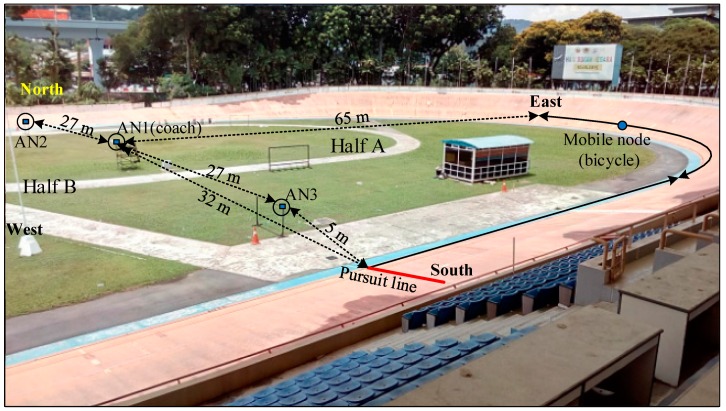
The outdoor experimental setting at the track cycling field (Cheras velodrome).

The proposed bicycle wireless sensor network (BWSN) topology is shown in Figure 2. The topology consisted of three ZigBee (XBee Series 2) anchor nodes, one ZigBee router node (RN), and two ZigBee sensor nodes (SNs). The RN and SNs, which moved along with the bicycle, are hereafter called the “mobile node”. The anchor nodes, namely AN1, AN2, and AN3, were mounted on a surface 1.5 m above the ground to avoid ground signal reflection or the Fresnel zone [44], while the router node was fixed under the bicycle seat, which was 0.85 m above the ground. AN1, which was located at the centre of the track cycling field, handled the reception of the bicycle parameters (i.e., speed, cadence, and torque) and sent a beacon signal to the mobile node for distance measurement. The data received by AN1 were displayed on the coach’s laptop or tablet to allow the coach to monitor the cyclist’s performance. AN1 could be connected to the coach’s laptop or any smart device, thus did not encounter any issue related to power consumption. The AN2 and AN3 were powered from the main AC source. Both AN2 and AN3 were not connected to AN1 but they operated independently. The anchor nodes AN2 and AN3 were 27 m away from AN1, as shown in Figure 3. The anchor nodes AN2 and AN3 were fixed at the north and south sides of the track to establish a consistent communication link with the bicycle node. AN2 and AN3 could not be located at the east or west sides because the mobile node on the track would have been at the farthest distance (130 m) from them, which would have resulted in a loss of communication. AN1, AN2 and AN3 sent a beacon signal to the mobile node for distance estimation purposes. Experiments were conducted in both outdoor and indoor environments.

### 3.1. Outdoor Experiment

The outdoor experiment was performed on a track cycling field (the Cheras velodrome, located in the middle of Kuala Lumpur, Malaysia). AN1 was located at the centre of the cycling field. The anchor nodes AN2 and AN3 were 27 m away from AN1 and the mobile node received the beacons from the anchor nodes to collect the RSSI data, as shown in Figure 3. The track cycling field area was 130 m × 65 m and 333 m long. The minimum and maximum distances between AN1 and the mobile node were 32 m (width of the velodrome) and 65 m (length of the velodrome), respectively, as measured from the centre of the field (Figure 3).

**Figure 4:**
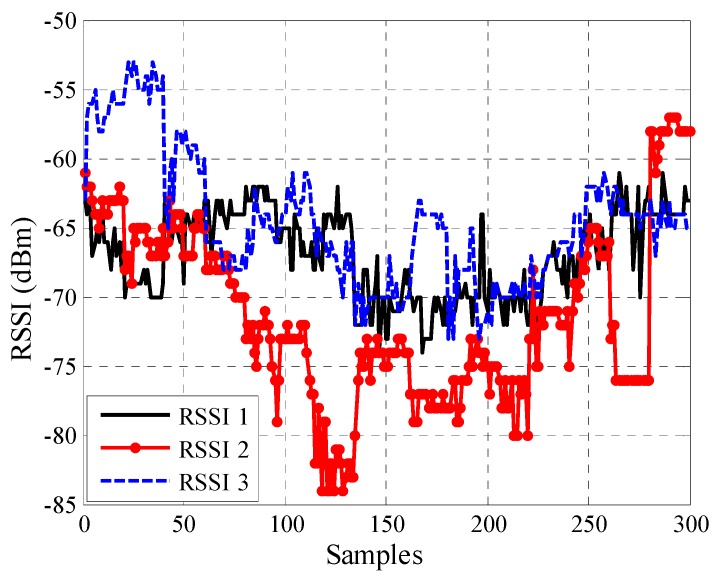
Measured RSSI values at the mobile node for three anchor nodes on a number of samples for each anchor node (300 samples) in the outdoor velodrome.

The bicycle track area was divided into two symmetrical halves (A and B), and the measurements were only performed for the first half (A). Half B was excluded due to the resemblance between the two halves. Half A was selected because it represented the starting line (pursuit line) for cycle training and competitions. The mobile node used the RSSI values to determine the distance between itself and AN1 according to the soft computing techniques. The RSSI was measured for 18 pre-defined positions in half A. A total of 20 samples were recorded at each position, with one sample per second. Each sample contained one data packet, and each data packet contained 34 bytes. Therefore, 900 samples (300 for each anchor node) of RSSI values of the three anchor nodes were collected and used for training, testing, and validating ANN and ANFIS to determine the distance to AN1. The collected RSSI values were used for training (70%) [45,46], testing (15%), and validating (15%) ANN and ANFIS to establish a relationship between the input RSSI values and the predicted physical distance. The measured RSSI values at the mobile node for three anchor nodes can be plotted with respect to the number of samples in the outdoor velodrome, as shown in Figure 4.

### 3.2. Indoor Experiment

**Figure 5:**
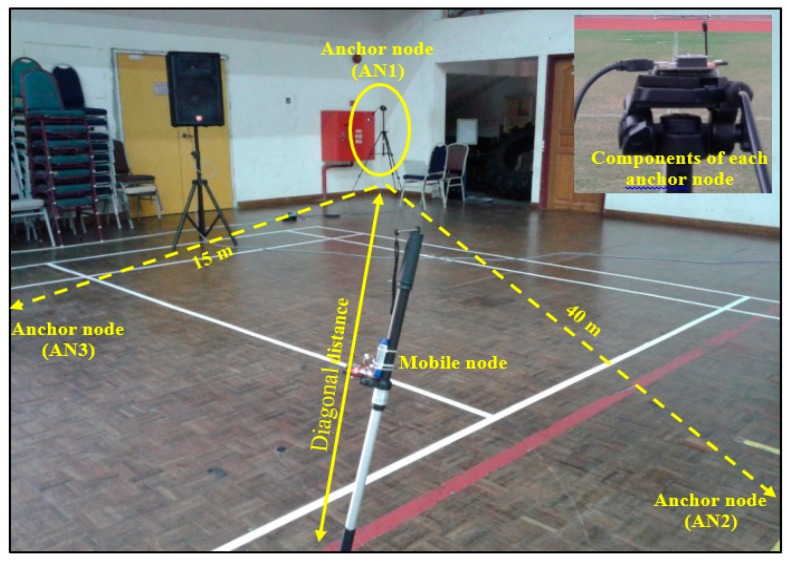
Sports hall (Dewan Gemilang) for the indoor experiment.

**Figure 6:**
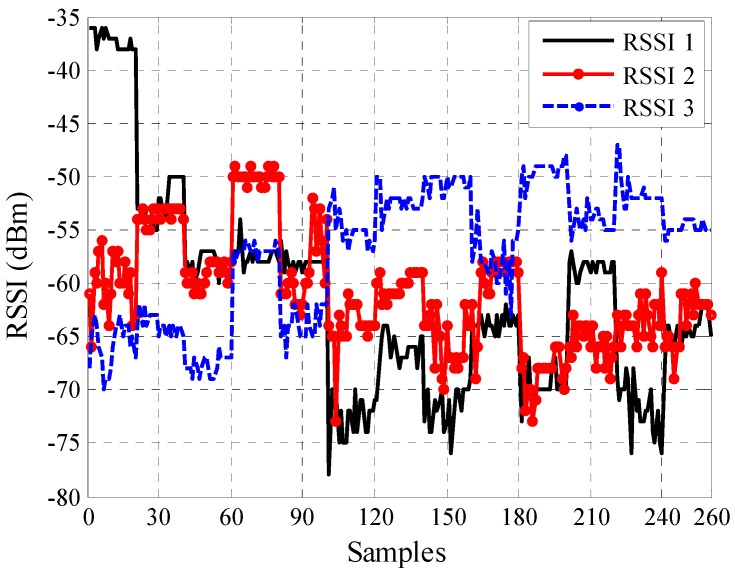
Measured RSSI values at the mobile node for the three anchor nodes with respect to a number of samples for each anchor node (260 samples) in the indoor velodrome.

The indoor experiment was performed in the sports hall (Dewan Gemilang) of the University Kebangsaan Malaysia, Bangi (Figure 5) to represent a quarter of the cycle track area as no indoor velodrome is available in the country. The velodrome geometry is symmetrical; therefore the considered indoor area was the closest resemblance to a quarter of an indoor velodrome. Due to area restrictions, the maximum number of points was reduced to 13 points. In addition, furniture and objects were excluded from the measurement area, which was almost similar to that of an actual velodrome. The building had an area of 36 m × 34 m. Given that the length of this building was not the same as that of a velodrome, a diagonal distance was considered to obtain the maximum distance between the mobile node and AN1 (coach), as shown in Figure 5. AN1 was located at one corner of the hall and anchor nodes AN2 and AN3 were 15 m and 40 m away from AN1, as shown in Figure 5. The RSSI values of AN1, AN2, and AN3 were collected by a mobile node from 13 pre-defined locations. A total of 20 samples were recorded at each position with one sample per second. Each sample contained one data packet, and each data packet contained 34 bytes. Therefore, 780 samples (260 for each anchor node) of RSSI values of the three anchor nodes were collected and used for training, testing, and validating ANN and ANFIS to determine the distance to AN1. The collected RSSI were used for training (70%), testing (15%), and validating (15%) ANN and ANFIS to establish a relationship between the input RSSI values and the predicted physical distance. The measured RSSI values at the mobile node for three anchor nodes can be plotted with respect to the number of samples in the indoor velodrome as shown in Figure 6.

## 4. Soft Computing-Based Localization Techniques

Soft computing techniques are employed to solve complex numerical optimization problems as well as non-linear and non-differentiable systems [47]. There are several categories of soft computing, for example ANFIS, ANN, Support Vector Machine (SVM), Fuzzy Logic (FL), and Optimization Algorithms (OA) [47]. Each category of soft computing also has a fine-grained set; for example, PSO, GRA, BSA, GA, BFA, Artificial Bee Colony (ABC), Ant Colony Optimization (ACO), and Differential Evolution (DE) are in the OA category. This paper focuses on ANFIS and the hybridization of PSO, GSA, and BSA with ANN backpropagation (BP) to accurately estimate the distance between bicycles on the cycle track and the coach. The selected soft computing techniques will be explained in detail in the following subsections.

### 4.1. ANFIS Techniques

**Figure 7:**
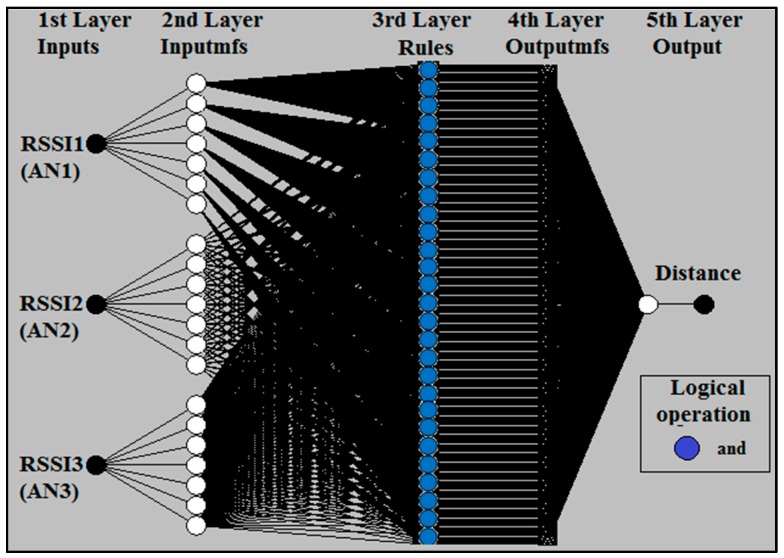
The adopted ANFIS structure for seven *gbellmfs*.

ANFIS is a technique for the evolution of self-organizing neuro-fuzzy systems [48]. ANFIS is employed to perform non-linear estimation algorithms, which in this case is the collected RSSI data. ANFIS has been used in research [26,49,50,51,52,53] to estimate the location of the nodes or distance between nodes in WSNs. In this work, the ANFIS structure shown in Figure 7 was adopted. The ANFIS input is the three RSSI values from three anchor nodes AN1, AN2, and AN3 for outdoor and indoor as shown in Figure 4 and Figure 6.

For each input, three, five, and seven inputs membership function (*mfs*) were trained and tested. Two types of membership function, namely the triangle membership (*trimf*) and generalized bell membership (*gbellmf*), were also used in ANFIS. Different numbers and types of membership function give different minimum distance error values. For training, testing, and validating ANFIS, a large number of RSSI samples are required. 900 samples (outdoor) and 780 samples (indoor) of RSSI values were used for training, testing, and validating ANFIS to accurately determine the distance between the bicycle on the track and the coach.

### 4.2. ANN Techniques

**Figure 8:**
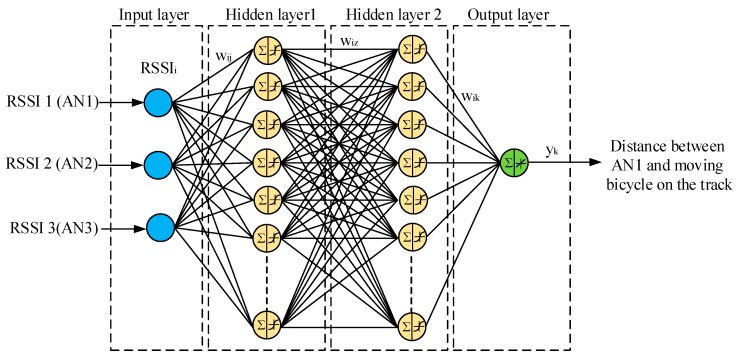
The architecture of the ANN algorithm.

ANN is an information processing system which has been developed as a generalized mathematical model of human biological nerves [54]. ANN-based localization techniques are able to model the complex mathematical relationship between the input variables (RSSI in current work) and target variable (distance). In this work, a BP neural network type and the Levenberg–Marquardt (LM) training algorithm were selected for training, testing, and validating. The LM training algorithm was selected because it gives minimum localization error, as proven in [55], in addition to its speed and efficiency. However, the LM algorithm requires a considerable amount of working memory [56]. For training, testing, and validating ANN, the same amounts of RSSI values which were used in ANFIS could be used for ANN to accurately determine the distance between the bicycle on the track and the coach for outdoor and indoor velodromes. In the ANN parameters the number of inputs, number of hidden layers, number of neurons in each hidden layer, learning rate, and the number of outputs must be determined before training, testing, and validating the data. In this paper, a four layer ANN architecture was built to determine the distance from the mobile node to the coach, based on the RSSI measurements of the anchor nodes. These layers included an: (i) input layer; (ii) first hidden layer; (iii) second hidden layer; and (iv) output layer, as shown in Figure 8. The input layer consisted of three RSSI values from the three anchor nodes, and the neurons in this layer only acted as a buffer for distributing the input signals *RSSI_i_* (*i* = 1, 2, 3, …, *n*) to the neurons in the first hidden layer. The input *RSSI_i_* were weighted against the strengths of particular connections *w_ij_* and summated by each neuron of the first hidden layer to pass the output of the first hidden layer to the neurons of the second hidden layer. The inputs of the second layer were weighted against the strengths of particular connections *w_iz_* and summated by each neuron of the second layer to calculate the output *y_k_* in the fourth layer. The first and second hidden layers used the *tansigmoidal* activation functions to cover all ranges of the negative RSSI values, whereas the output layer employed the *linear* activation functions to cover the positive values of distance. The first and second hidden layers consisted of number of neurons in each hidden layer. The number of neurons in each hidden layer and learning rate were selected based on the optimization algorithms (PSO, GSA, and BSA) because this parameter selection was not secure and subject to the trial-and-error method, which does not always provide the optimum solution. The PSO, GSA, and BSA algorithms address such a problem by determining the best number of neurons in each hidden layer and the optimum learning rate of ANN. Thus, the performance of ANN can be improved. In this case, these algorithms could be hybridized with ANN to form three different algorithms, which were known as the hybrid PSO-ANN algorithm, hybrid GSA-ANN algorithm, and hybrid BSA-ANN algorithm through which ANN was able to achieve a minimum distance error.

### 4.3. Heuristic Algorithms

Heuristic algorithms are a technique which tries to find a good solution (near optimal) at a sensible computational cost without the ability to undertake either optimality or feasibility, or even in several cases to clarify how close to the optimum solution [57]. Because the classical WSN localization methods provided a high localization error, ANN was adopted in this paper to improve the estimated localization error. Due to the learning capabilities and flexible modelling of ANN, it is possible to perform lesser errors in determining the distance between the coach and the bicycle on the cycle track without detailed knowledge of the surroundings. With sufficient ANN parameters and a large amount of RSSI values, ANN is capable of representing the relationship between inputs (RSSI) and output (distance between coach and the bicycle). The heuristic algorithms, such as PSO, GSA, and BSA, were hybridized with ANN to determine the optimum ANN parameters (i.e., number of neurons in each hidden layer and the learning rate). Selecting these parameters is not secure and subject to trial and error, which in return gives a high distance estimation error.

Using the parameter settings of heuristic algorithms as shown in Table 1, we executed the PSO, GSA, and BSA algorithms and obtained the fitness function for 10, 20, 30, 40, and 50 population sizes. Several population sizes were implemented to allow each algorithm to select the population that could achieve the minimum fitness function and elapsed time. Nevertheless, there is no exact algorithm to provide an accurate result for all optimization problems. Some optimization algorithms provide a better solution for some specific problems compared to others. From the execution of the algorithms, it was revealed that the hybrid GSA-ANN achieved minimum errors compared to the other algorithms. Consequently, a hybrid GSA-ANN was considered in the current work. The training processes of ANN were repeated several times using a large number of epochs (i.e., 1000 iterations) until the error between the actual and predicted distances reached the minimum.

The GSA algorithm was proposed in 2009 by Rashedi et al. [57]. This algorithm relies on Newtonian gravity: ‘‘Every particle in the universe attracts every other particle with a force that is directly proportional to the product of their masses and inversely proportional to the square of the distance between them” [57]. The GSA mathematical principle is based on the law of Newtonian gravity and the laws of motion as in the following:

(1) $$F=G\;\frac{M_{1}M_{2}}{R^{2}}$$

where *F* is the gravitational force, *R* is the distance between the first particles mass (*M*_1_) and second particles mass (*M*_2_), and *G* is the constant value of the gravitational. Newton’s Second Law states that acceleration *a* is inversely proportional to mass *M* and directly proportional to force *F* as follows:

(2) $$a=\;\frac{F}{M}$$

Because of the effect of declining gravity, the real value of the “gravitational constant (*G*)” depends on the real age of the universe. Equation (3) provides the decrease of the gravitational constant with age [58]:

(3) $$G\left(t\right)=G\left(t_{0}\right)\times {\left(\frac{t_{0}}{t}\right)}^{\beta }\;\beta <1\;$$

where *G*(*t*) is the gravitational constant at time *t* and *G*(*t*_0_) is the gravitational constant at the first cosmic quantum-interval of time *t*_0_ [58].

The positions of the agents are initialized (i.e., the masses are randomly selected within the given search interval). The position of the *i^th^* agent can be defined by:

(4) $$X_{i}=\left(X_{i}^{1},\ldots \ldots ,X_{i}^{d},\ldots \ldots ,X_{i}^{k}\right),\;for\;i=1,2,\ldots ,N\;$$

where *N* is the number of agents, $X_{i}^{d}$ is the position of *i^th^* agent in the *d^th^* dimension and *k* is the space dimension. To compute the fitness function of GSA, a root mean square error (RMSE) can be used to determine the best and the worst fit for each iteration. The computations aim to minimize the problems and determine the masses of each agent as follows [59]:

(5) $$RMSE=\sqrt{\frac{1}{n}\overset{n}{\underset{i=1}{\sum }}e^{2}}\;\;$$

(6) $$best\left(t\right)=\underset{\text{ j}\in \left\{1,\ldots ,N\right\}}{\min}\;{fit}_{j}\left(t\right)$$

(7) $$worst\left(t\right)=\;\underset{\text{ j}\in \left\{1,\ldots ,N\right\}}{\max}\;{fit}_{j}\left(t\right)$$

(8) $$m_{i}\left(t\right)=\frac{fit_{i}\left(t\right)-Worst\left(t\right)}{best\left(t\right)-Worst\left(t\right)}$$

(9) $$M_{i}\left(t\right)=\frac{m_{i}\left(t\right)}{\sum_{j=1}^{N}m_{i}\left(t\right)}$$

where *e* is the estimated distance error and *n* is the number of samples. The actual distance was obtained based on measurement, whereas the estimated distance was obtained using the hybrid GSA-ANN. The gravitational constant G at iteration *t* was computed as follows:

(10) $$G\left(t\right)=G_{0}e^{\left(-\alpha t/T\right)}$$

The total force computation in different directions in the *i^th^* agent, the velocity and acceleration calculation, and the position of the agents in the next iteration are as follows:

(11) $$F_{ij}^{d}\left(t\right)=G\left(t\right)\frac{M_{pi}\times M_{aj}}{R_{ij}+\varepsilon }\left(X_{j}^{d}\left(t\right)-X_{i}^{d}\left(t\right)\right)$$

(12) $$F_{i}^{d}\left(t\right)=\underset{j\in Kbest,j\neq i}{\sum }{\text{rand}}_{j}F_{ij}^{d}\left(t\right)$$

(13) $$a_{i}^{d}\left(t\right)=\frac{F_{i}^{d}\left(t\right)}{M_{i}\left(t\right)}$$

(14) $$v_{i}^{d}\left(t+1\right)=rand_{i}\times v_{i}^{d}\left(t\right)+a_{i}^{d}\left(t\right)$$

(15) $$x_{i}^{d}\left(t+1\right)=x_{i}^{d}\left(t\right)+v_{i}^{d}\left(t+1\right)$$

**Figure 9:**
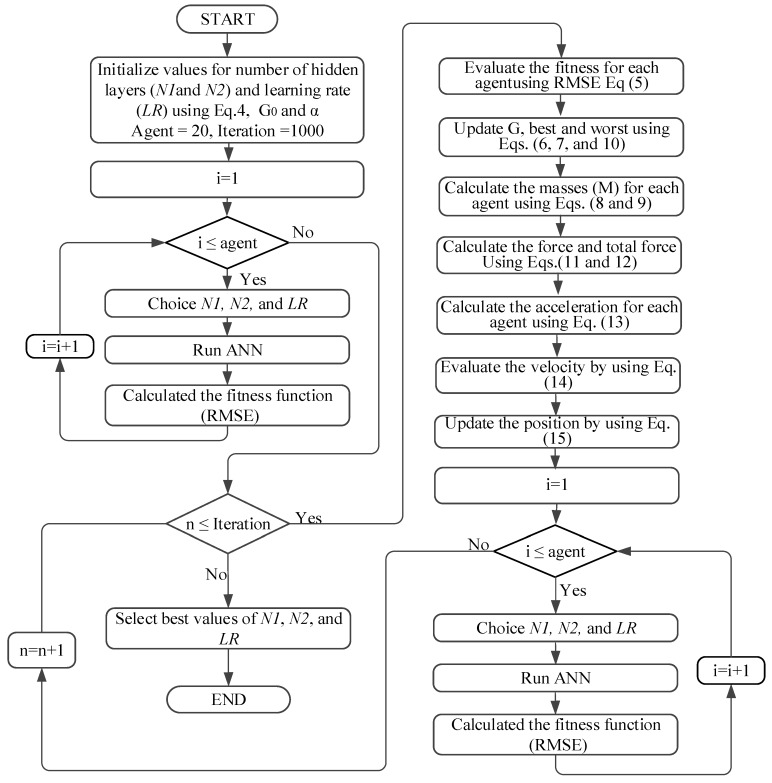
Flow chart of hybrid GSA-ANN.

The details of the operation of the hybrid GSA-ANN based on the previous equations are shown in the flow chart in Figure 9.

## 5. Results and Discussion

In this section the results of the soft computing techniques will be discussed in terms of distance estimation accuracy. The ANFIS results will be presented first, followed by those of the hybrid PSO-ANN, hybrid GSA-ANN, and hybrid BSA-ANN. The outcomes of the hybrid GSA-ANN will be considered for comparison with the previous works because it achieved the minimum distance error relative to the other soft computing techniques.

### 5.1. ANFIS Techniques

**Figure 10:**
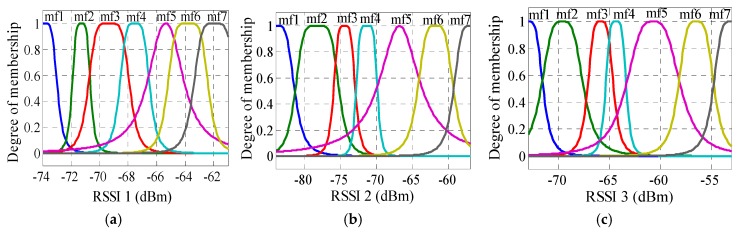
ANFIS seven *gbellmfs* input of outdoor velodrome (**a**) RSSI 1; (**b**) RSSI 2; and (**c**) RSSI 3.

**Figure 11:**
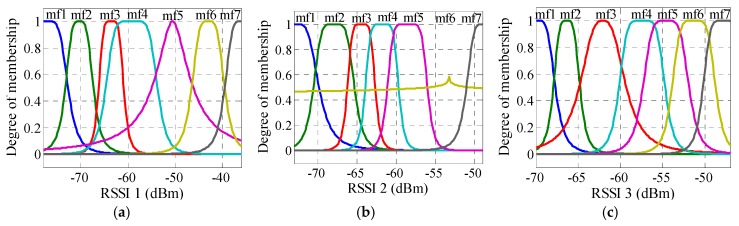
ANFIS seven *gbellmfs* input of indoor velodrome (**a**) RSSI 1; (**b**) RSSI 2; and (**c**) RSSI 3.

**Figure 12:**
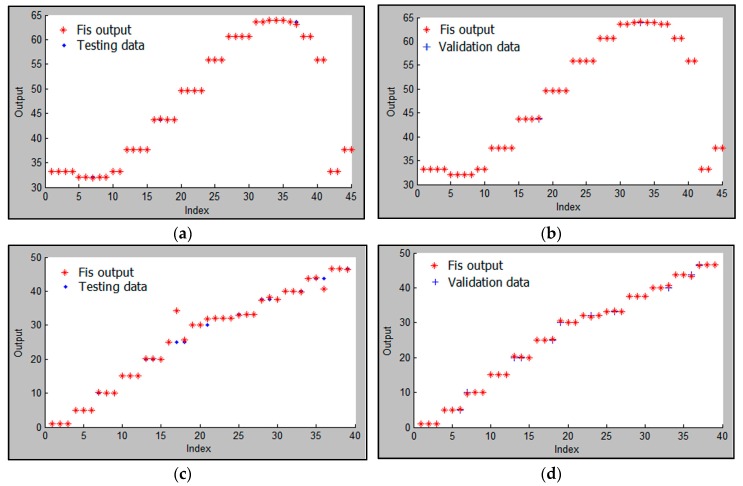
Comparison of transient characteristics for ANFIS (**a**) testing and (**b**) validation data for the outdoor velodrome and (**c**) testing and (**d**) validation for the indoor velodrome.

As previously mentioned, the collected RSSI values were used for training and testing ANFIS (70% for training, 15% for testing, and 15% for validating) to establish a relationship between the input RSSI values and the output physical distances. As a result, the distance error was obtained for different numbers and types of the membership function for outdoor and indoor, as shown in Table 2. The table shows MAE and RMSE, whereby the minimum error occurred when seven membership functions were selected for each input. The distance estimation accuracy is poor for the indoor scenario case, which is expected due to the presence of multipath scatters and reflectors that dominate an indoor environment. In addition, the *gbellmf* type is better than *trimf* for both outdoor and indoor. Therefore, seven *gbellmfs* were considered in this study for outdoor and indoor velodromes. For each ANFIS input, the membership function is shown in Figure 10 and Figure 11 for outdoor and indoor, respectively. Figure 12 shows the comparison transient characteristics for seven *gbellmfs* after testing and validating data for outdoor and indoor velodromes. The figure shows that there is no significant difference between the estimated and the actual distance for outdoor and indoor velodromes. The red points represent the estimated distance (FIS out), whereas the blue points and plus signs are the actual distance (testing and validating data). For outdoor environments, most of the estimated distances matched the actual distances, as can be seen in Figure 12a (testing data) and Figure 12b (validation data). However, for indoor environments the difference between the estimated distance and the actual distance was very small, as shown in Figure 12c (testing data) and Figure 12d (validation data). This is due to the same reason mentioned previously, i.e., the presence of multipath scatters and reflectors.

### 5.2. Hybrid Heuristic Algorithms-ANN Techniques

**Figure 13:**
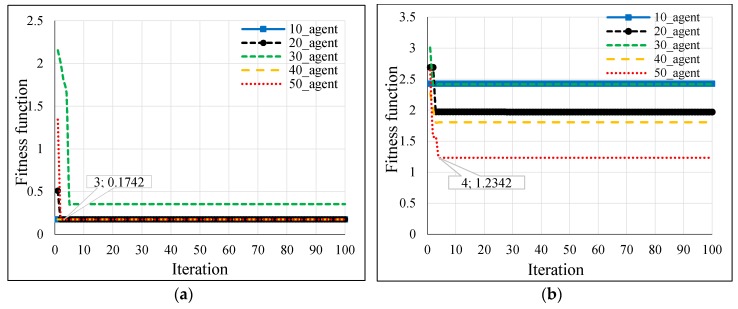
GSA fitness function versus iteration for (**a**) outdoor and (**b**) indoor velodromes.

**Figure 14:**
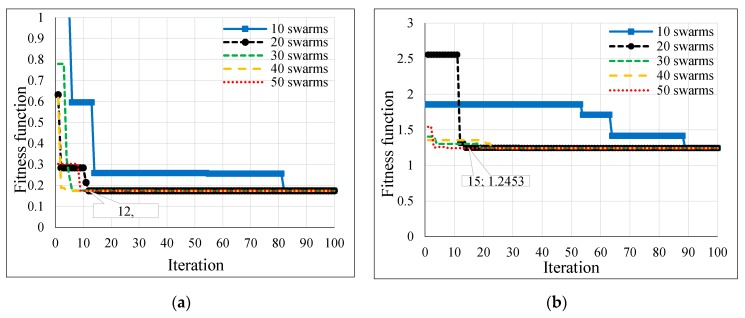
PSO fitness function versus iteration for (**a**) outdoor and (**b**) indoor velodromes.

**Figure 15:**
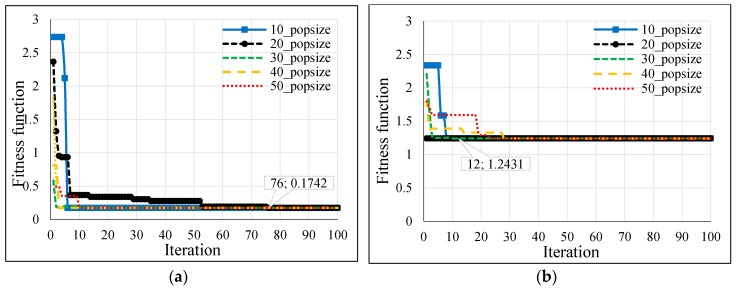
BSA fitness function versus iteration for (**a**) outdoor and (**b**) indoor velodromes.

Several population sizes were simulated in Matlab for the hybrid GSA-ANN, hybrid PSO-ANN, and hybrid BSA-ANN to allow each heuristic algorithm to select the optimum number of neurons in each hidden layer and learning rate of ANN. Thereby, the minimum fitness function could be achieved. Table 3 shows the neurons of the hidden layers (N1 and N2) and LR, which were obtained from the implementation of each heuristic algorithm in Matlab based on different population sizes for outdoor and indoor velodromes. The fitness functions of the hybrid GSA-ANN, hybrid PSO-ANN, and hybrid BSA-ANN for different population sizes are shown in Figure 13, Figure 14 and Figure 15, respectively, for outdoor and indoor environments.

**Figure 16:**
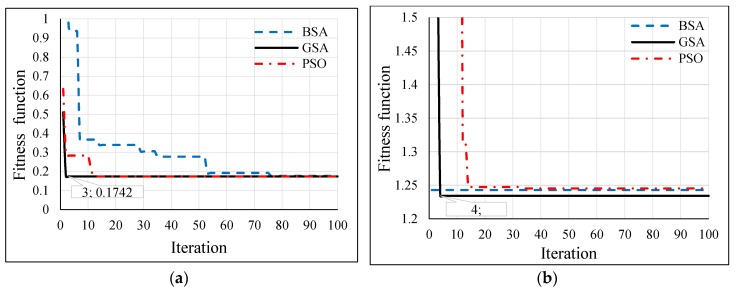
Fitness function comparison of GSA, PSO, and BSA for (**a**) outdoor and (**b**) indoor velodromes.

Once the adopted population size was tested for each heuristic algorithm, it was found that the 20 population size gave the minimum fitness function (based on RMSE) for all heuristic algorithms for the outdoor velodrome, whereas the population size was 20 for the hybrid PSO-ANN and hybrid BSA-ANN and 50 for the hybrid GSA-ANN, which gave the minimum fitness function for the indoor velodrome. On the other hand, GSA-ANN achieved the minimum fitness function compared to the other heuristic algorithms for outdoor and indoor. GSA-ANN was able to reach the best fitness function at 20 (outdoor) and 50 (indoor) population sizes, as shown in Figure 16a,b, respectively. Based on the outcomes of the hybrid GSA-ANN, ANN was trained, tested, and validated using the parameters that achieved the minimum fitness function (i.e., N1 = N2 = 17, and LR = 0.8004 for outdoor and N1 = 13, N2 = 10, and LR = 0.535 for indoor) as shown in Table 3. These parameters improved the ANN operation during the training, testing, and validation phases, which resulted in a high distance estimation accuracy.

**Figure 17:**
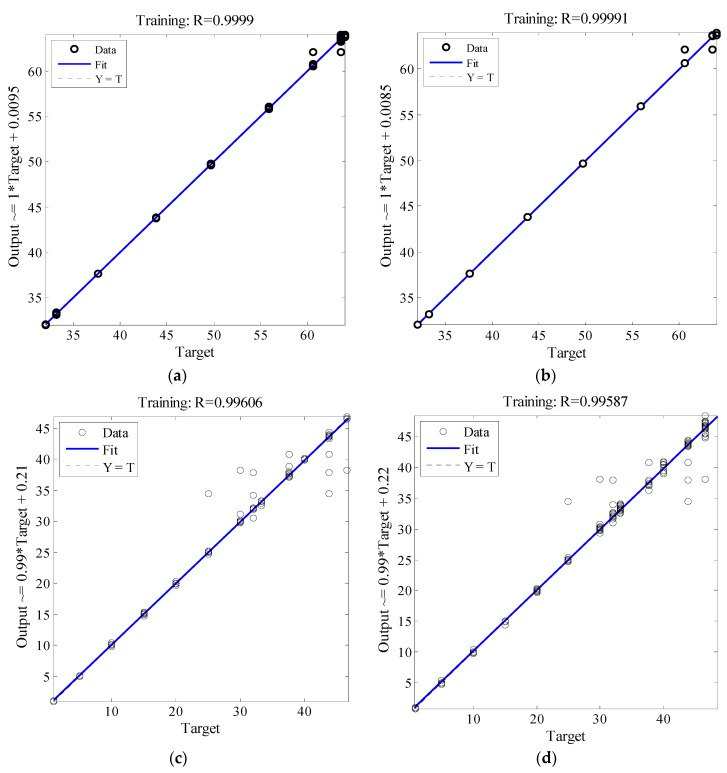
The performance of the hybrid GSA-ANN algorithm for (**a**) testing and (**b**) validation data for the outdoor velodrome and (**c**) testing and (**d**) validation for the indoor velodrome.

Figure 17 shows the testing and validation data of ANN. The actual distance (i.e., the target on the *x*-axis) for the outdoor and indoor velodrome is plotted against the estimated distance (i.e., output on the *y*-axis). The regression coefficient (R) of determination between the actual and estimated distance is a good indicator for the investigation of the prediction performance of the hybrid GSA-ANN algorithm. From Figure 17a,b for the outdoor velodrome, the *R* values were 0.9999 (testing) and 0.99991 (validation). For Figure 17c,d for the indoor velodrome, the *R* values were 0.99606 (testing) and 0.99587 (validation). The regression coefficient results suggested a close agreement between the actual and estimated distances.

The hybrid GSA-ANN was used to optimize the ANN operation by selecting the optimum values of neurons in each hidden layer and learning rate. Although GSA is a famous optimization technique [60] it has not been hybridized with ANN in the previous literature to address the localization problems in WSNs. In the current work, the GSA technique outperforms PSO and BSA, whereby it achieves a fast convergence with a lesser number of iterations, i.e., 3 and 4 iterations and a minimum RMSE of 0.1742 and 1.2342 for outdoor and indoor, as shown in Figure 16a,b, respectively. Consequently, a lesser computational time was accomplished for convergence through this algorithm relative to other adopted algorithms. ANN was trained, tested, and validated offline using the measured RSSI values as inputs and the actual distance between the coach and the bicycle on the cycle track as output. Since the training, testing, and validating of ANN was done offline and not implemented in real time, there were no issues related to delay characteristics. One advantage of the proposed hybrid GSA-ANN is that it does not need training for any new distance estimation when applied in real time. Once the model is trained offline, any new inputs of RSSI values from the anchor nodes can be given in real time to the neural network and the corresponding distance estimation can be obtained. However, the training time in the offline phase was 23 s for BSA-ANN and 33 s for GSA-ANN and PSO-ANN. The training time of the adopted hybrid heuristic algorithms have performed the Bayesian Regularization (BR) algorithm of ANN in research work [61], whereby the training time was 751 s.

## 6. Results Comparison

**Figure 18:**
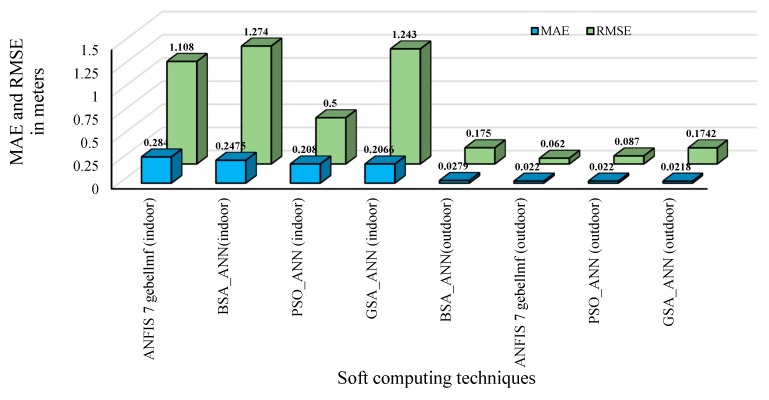
Comparison of the distance estimation error for adopted soft computing techniques for outdoor and indoor velodromes.

Figure 18 shows the comparison of the distance estimation error between the soft computing techniques considered in the current work in terms of MAE and RMSE for outdoor and indoor velodromes. Both RMSE and MAE can be used when GSA is hybridized with ANN. RMSE and MAE can be employed together to detect the variation in the errors in a set of predictions. MAE will always be smaller than or equal to RMSE; the greater the difference between them, the greater the variance in the discrete errors in the sample. If MAE is equal to RMSE, then all the errors have the same magnitude.

It can be observed that there was a small difference between MAE and RMSE for the case of outdoor, as shown in Figure 18, which was expected due to the lesser signal attenuation. In contrast, the gap between RMSE and MAE was very huge, suggesting that the variance error in terms of accuracy is large for indoor samples. The figure shows that MAE and RMSE for outdoor are better than indoor for all adopted techniques, whereas the hybrid GSA-ANN was best for outdoor and indoor velodromes. MAE of hybrid GSA-ANN was improved by 27% and 16% relative to ANFIS seven *gbellmfs* and hybrid BSA-ANN, respectively, for the indoor velodrome, whereas the MAE of hybrid GSA-ANN was improved by 21% relative to the hybrid BSA-ANN for the outdoor velodrome.

**Figure 19:**
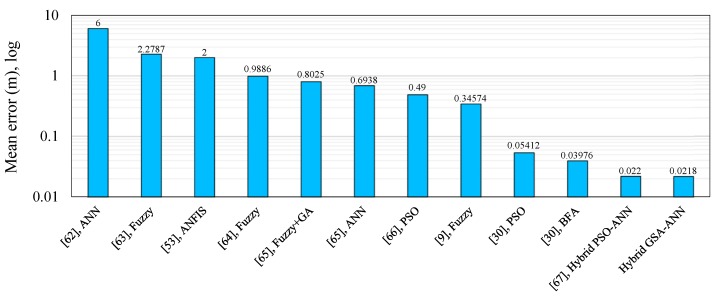
Comparison of GSA-ANN techniques with previous works for outdoor environments.

**Figure 20:**
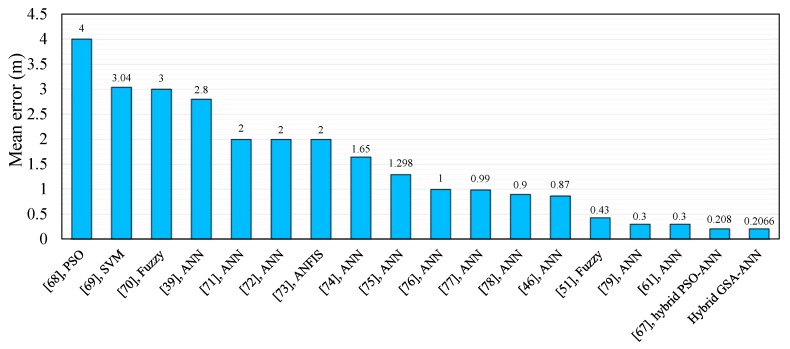
Comparison of GSA-ANN techniques with previous works for indoor environments.

The hybrid GSA–ANN algorithm can be compared with previous works [9,30,39,46,51,53,61,62,63,64,65,66,67,68,69,70,71,72,73,74,75,76,77,78,79] in terms of localization or distance error to validate our proposed system. Similar studies based on different soft computing techniques were considered for the purposes of comparison. The RSSI performance metric was employed as the input, whereas the location of the target node or the distance between the nodes in the network was used as the output. Most of these studies used ANN and Fuzzy Logic techniques to improve localization accuracy, and some of them used optimization algorithms such as PSO or hybrid techniques. Figure 19 (outdoor) and Figure 20 (indoor) show the comparison of the hybrid GSA-ANN with those works. The mean localization or distance error is considered a performance metric for the comparison between our work and previous studies. Our proposed hybrid GSA–ANN algorithm outperforms the algorithms or techniques of the other studies with its average distance errors of 0.0218 m and 0.2066 m for outdoor and indoor velodromes, respectively.

## 7. Conclusions

Two soft computing techniques for WSN distance estimation were presented in this paper for outdoor and indoor velodromes. These techniques aimed to determine the distance between the bicycle position while moving on the cycle track and the coach. The first method was based on ANFIS, whereas the second approach adopted heuristic algorithms such as GSA, PSO, and BSA hybridized with ANN. The ANN algorithm was further improved by combining with GSA, PSO, and BSA to select the optimum number of neurons in the adopted two hidden layers and to select the optimum learning rate of ANN. This resulted in an improvement of the distance error between the bicycle and the coach. The soft computing techniques were compared with each other to select the best algorithm that gave the minimum distance error. The results indicated that the hybrid GSA-ANN was more convenient than the other algorithms adopted in this work in terms of distance estimation accuracy. The comparison results showed that the MAE of the hybrid GSA-ANN outperformed the previous works for both outdoor and indoor environments. Therefore, GSA-ANN is suitable in both indoor and outdoor environments and can be applied to any static or mobile WSN node.

The limitation of this study lies in the possibility of implementing the hybrid GSA-ANN in real-time as the implementation of ANN requires a considerable amount of memory. The huge computation memory comes at the expense of limited memory size and processor speed of the microcontrollers. In such a case, the mobile node (bicycle) requires a microcontroller with a high speed and large memory size such as an Arduino Due. However, using an Arduino Due leads to high power consumption, large size and extra weight, all of which are considered critical issues in bicycle sensor nodes. The high level of power consumption will eventually reduce the battery life of the sensor nodes. In addition, the extra size and weight on the bike increases aerodynamic resistance, which consequently reduces the bike’s speed and induces fatigue in the athlete during cycling. These considerations are critical in competitive events. It is expected that once quantum computing is in place and Moore’s Law has achieved its saturation in future, the use of high computing will be possible for small sensor applications.

## Figures and Tables

**Table 1:** Parameters of the heuristic algorithms.

Parameter	PSO	GSA	BSA
Population size 3	10, 20, 30, 40, and 50	10, 20, 30, 40, and 50	10, 20, 30, 40, and 50
Iteration	100	100	100
c1 and c2	1.494	-	-
*w*	0.7	-	-
*F*	-	-	3
G_0_ and α	-	1, 0.2	-

**Table 2:** Comparison of distance estimation accuracy based on ANFIS.

ANFIS Method	Outdoor		Indoor	
	MAE (m)	RMSE (m)	MAE (m)	RMSE (m)
3 *trimf*	2.486	3.938	3.581	4.725
5 *trimf*	1.588	2.647	1.91	3.05
7 *trimf*	0.3634	0.907	1.004	2.107
3 *gbellmf*	2.335	3.713	2.927	3.978
5 *gbellmf*	0.4695	0.796	1.4269	2.476
7 *gbellmf*	0.022	0.062	0.284	1.108

**Table 3:** Neurons in each hidden layer and learning rate of ANN based on heuristic algorithms for different population sizes.

Population Size	Parameters	GSA-ANN		PSO-ANN		BSA-ANN	
		Outdoor	Indoor	Outdoor	Indoor	Outdoor	Indoor
10	N1	19	15	12	9	14	11
	N2	19	17	18	16	19	18
	LR	0.2541	0.3429	0.1877	0.4477	0.2505	0.6652
20	N1	17	10	18	7	10	15
	N2	17	16	16	18	19	18
	LR	0.8004	0.7394	0.0709	0.5864	0.3492	0.223
30	N1	6	14	17	14	15	15
	N2	12	11	18	14	19	19
	LR	0.5864	0.4764	0.4533	0.5027	0.8871	0.6608
40	N1	18	10	17	15	17	8
	N2	16	14	16	16	17	17
	LR	0.5947	0.5152	0.6127	0.4206	0.7139	0.9743
50	N1	14	13	11	16	16	17
	N2	13	10	18	19	18	19
	LR	0.5487	0.535	0.9407	0.7194	0.4737	0.4655

N1: number of neurons in hidden layer 1, N2: number of neurons in hidden layer 1, and LR: learning rate of ANN.
